# A Recent SARS-CoV-2 Infection Enhances Antibody-Dependent Cellular Cytotoxicity against Several Omicron Subvariants following a Fourth mRNA Vaccine Dose

**DOI:** 10.3390/v15061274

**Published:** 2023-05-29

**Authors:** Guillaume Beaudoin-Bussières, Alexandra Tauzin, Katrina Dionne, Gabrielle Gendron-Lepage, Halima Medjahed, Josée Perreault, Inès Levade, Laila Alfadhli, Yuxia Bo, Renée Bazin, Marceline Côté, Andrés Finzi

**Affiliations:** 1 Centre de Recherche du CHUM, Montréal, QC H2X 0A9, Canada; 2 Département de Microbiologie, Infectiologie et Immunologie, Université de Montréal, Montréal, QC H2X 0A9, Canada; 3 Héma-Québec, Affaires Médicales et Innovation, Québec, QC G1V 5C3, Canadarenee.bazin@hema-quebec.qc.ca (R.B.); 4 Laboratoire de Santé Publique du Québec, Institut National de Santé Publique du Québec, Sainte-Anne-de-Bellevue, QC H9X 3R5, Canada; 5 Department of Biochemistry, Microbiology and Immunology, and Center for Infection, Immunity and Inflammation, University of Ottawa, Ottawa, ON K1H 8M5, Canadabyuxia@uottawa.ca (Y.B.)

**Keywords:** coronavirus, COVID-19, SARS-CoV-2, spike glycoproteins, variants of concern, omicron, ADCC, Fc-effector responses, hybrid immunity

## Abstract

Since the beginning of the SARS-CoV-2 pandemic, several variants of concern (VOCs), such as the Alpha, Beta, Gamma, Delta and Omicron variants, have arisen and spread worldwide. Today, the predominant circulating subvariants are sublineages of the Omicron variant, which have more than 30 mutations in their Spike glycoprotein compared to the ancestral strain. The Omicron subvariants were significantly less recognized and neutralized by antibodies from vaccinated individuals. This resulted in a surge in the number of infections, and booster shots were recommended to improve responses against these variants. While most studies mainly measured the neutralizing activity against variants, we and others previously reported that Fc-effector functions, including antibody-dependent cellular cytotoxicity (ADCC), play an important role in humoral responses against SARS-CoV-2. In this study, we analyzed Spike recognition and ADCC activity against several Omicron subvariants by generating cell lines expressing different Omicron subvariant Spikes. We tested these responses in a cohort of donors, who were recently infected or not, before and after a fourth dose of mRNA vaccine. We showed that ADCC activity is less affected than neutralization by the antigenic shift of the tested Omicron subvariant Spikes. Moreover, we found that individuals with a history of recent infection have higher antibody binding and ADCC activity against all Omicron subvariants than people who were not recently infected. With an increase in the number of reinfections, this study helps better understand Fc-effector responses in the context of hybrid immunity.

## 1. Introduction

Near the end of 2019, a new coronavirus was detected in Wuhan, China, which was named severe acute respiratory syndrome coronavirus 2 (SARS-CoV-2) [1,2,3]. Quickly after the discovery of this virus, multiple infections were reported all around the globe and millions were infected within a few months. To counter this pandemic, several vaccine platforms were developed at an unprecedented speed [4,5]. With the large-scale deployment of these vaccines and the rapid propagation of SARS-CoV-2, a significant portion of the population developed an immunity against this virus. However, this immunity led to an increased immune pressure and contributed to the rise of multiple variants under monitoring (VUMs), variants of interest (VOIs) and variants of concern (VOCs), depending on their transmissibility and prevalence, capacity to evade humoral responses/previously generated immunity, severity of disease and reduced effectiveness of treatments and diagnostics.

To evaluate the ability of the immune response induced by infection and/or vaccination to protect against SARS-CoV-2 infections (especially against the developing VUMs, VOIs and VOCs), multiple studies measured the antibody response against the SARS-CoV-2 Spike. Antibodies have multiple functions that can help in viral clearance. On the one hand, they can bind the Spike glycoproteins at the surface of the virions and restrict their ability to infect cells if they prevent the interaction of the Spike with the angiotensin-converting enzyme 2 (ACE2) host receptor [6] or if they prevent further downstream conformational changes [7,8]. The robustness of the neutralizing response against different variants has been extensively studied and is still under intense scrutiny with the advent of each new VOC [9,10,11,12,13,14,15,16,17,18]. It was previously shown that all VOCs (Alpha, Beta, Gamma, Delta and Omicron variants) were more resistant to neutralization by plasma from infected and/or vaccinated individuals [9,10,11,12,19,20], with the Omicron variant being the most resistant to neutralization [13,14,15,19,20]. This variant quickly replaced the other VOCs and gave rise to multiple sublineages which are increasingly more resistant to neutralization [16,18,21,22,23,24,25].

On the other hand, antibodies induced by SARS-CoV-2 infection/vaccination can also bind the Spikes expressed at the surface of infected cells [26]. This can lead to Fc-effector responses, including complement-dependent cytotoxicity (CDC), antibody-dependent cellular phagocytosis (ADCP) and antibody-dependent cellular cytotoxicity (ADCC), which result in the elimination of the infected cells. While the capacity of new variants to evade the neutralizing response is being extensively studied [9,10,11,12,13,14,15,16,18,19,20,21,22,23,24,25], Fc-effector responses, such as ADCC, have been less investigated. Nevertheless, some lines of evidence point towards Fc-effector functions having an important role in combating SARS-CoV-2 infection. First, a significant portion (25–45%) of individuals who resolved a SARS-CoV-2 infection (before vaccination) had a low or even undetectable SARS-CoV-2 neutralizing activity, while maintaining high antibody titers [3,27,28,29,30,31,32]. Second, while a significant loss in the neutralizing ability of the AstraZeneca vaccine against the Alpha variant was measured, it remained efficacious against this VOC [33]. Also, effectiveness of a first dose of vaccine beginning after two weeks reached >90% in the absence of neutralizing activity but with ADCC readily detected [34,35]. Third, other studies found that compromised Fc-effector responses were significantly associated with mortality in acutely infected individuals [36,37]. Fourth, in animal models, neutralizing antibodies required Fc-effector responses to enable complete viral clearance and have optimal therapeutic efficacy [38,39]. In addition, Fc-effector responses alone delayed virus spread, neuroinvasion and death [40]. These lines of evidence show that Fc-effector responses have to be considered when investigating protective humoral responses against emerging VOCs.

The emergence of multiple Omicron subvariants led to successive waves of infections. Because of the significant rise in infections and hospitalizations, a third and fourth dose of vaccine was recommended for the general population. In this study, we investigated the ADCC responses against the D614G strain and the BA.1, BA.2, BA.2.12.1 and BA.4/5 Omicron subvariants after a fourth dose of mRNA vaccine. Since a large part of the population has been recently infected, we also investigated the effect of hybrid immunity on the Fc-effector response against these Omicron subvariants. To investigate ADCC responses, we constructed CEM.NKr cell lines expressing the D614G, BA.1, BA.2, BA.2.12.1 or BA.4/5 Spikes. These cell lines were used to measure the ADCC activity of plasma samples from a cohort of healthcare workers collected after the third and fourth dose of vaccine. The cohort was separated into two groups depending on the history of recent infection (based on the variation in their anti-nucleocapsid (N) antibody level, as recently reported [41]). We observed that a recent infection significantly enhanced antibody production and ADCC. Furthermore, while the neutralizing activity has been reported to rapidly decrease over time [42,43], ADCC remained more stable.

## 2. Materials and Methods

### 2.1. Ethics Statement

The study was conducted in accordance with the Declaration of Helsinki in terms of informed consent and approval by an appropriate board. The protocol was approved by the Ethics Committee of the CHUM (19.381, approved on 28 February 2022) and Héma-Québec (2022-016, approved on 7 October 2022).

### 2.2. Human Subjects and Plasma Samples

**Figure 1:**
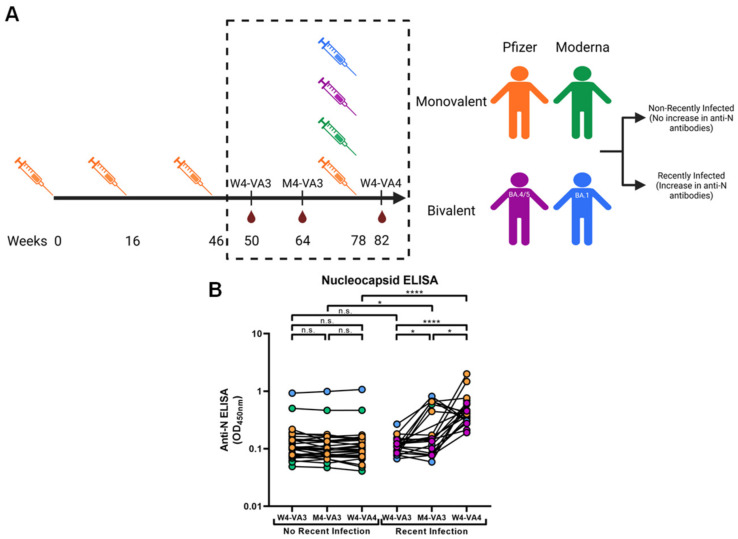
Cohort design and anti-N evaluation. (**A**) SARS-CoV-2 vaccine cohort timeline for administration of the vaccine doses and sampling. The syringes on the timeline represent the time of vaccine administration and blood drops represent the time of blood sampling. Individuals which had their fourth dose of vaccine being with Pfizer monovalent, Moderna monovalent, Pfizer bivalent and Moderna bivalent are represented by orange, green, purple and blue syringes, respectively. (**B**) Anti-N levels measured following the third and fourth dose of vaccine. This anti-N level was measured in the plasma of every individual donor at each timepoint available by ELISA. Donors were separated in non-recently infected individuals (n = 26) and recently infected individuals (n = 20) based on an increase in anti-N levels between W4-VA3 and M4-VA3 or between M4-VA3 and W4-VA4. Statistical significance within groups (paired *t*-test or Wilcoxon test) or between groups (unpaired *t*-test or Mann–Whitney test) was evaluated using the appropriate parametric or non-parametric statistical test based on the normal distribution of the data (n.s.: non-significant; *: *p* < 0.05; ****: *p* < 0.0001).

The cohort characteristics are provided in Table 1 and time of vaccination/blood sample collection are illustrated in Figure 1A. Briefly, the study was conducted in 46 individuals (22 males and 24 females; age range: 24–84 years). Plasma samples were recovered at an average of 3 to 4 weeks following the third dose (W4-VA3), 4 months following the third dose (M4-VA3) and 3 to 4 weeks following the fourth dose (W4-VA4). These plasma samples were either recovered from whole blood or directly obtained from the PlasCoV biobank [44], heat-inactivated for 1h at 56 °C and stored at −80 °C until use in subsequent experiments. Pre-pandemic plasma samples were used as negative controls in cytometry and ADCC assays (data not shown) for each experiment. In total, 20 of the participants had a recent breakthrough infection with an Omicron subvariant (8 males and 12 females; age range: 24–67 years), i.e., as determined by the increase in anti-N levels between W4-VA3 and M4-VA3 or between M4-VA3 and W4-VA4 (ratio M4-VA3/W4-VA3 and/or W4-VA4/M4-VA3 higher than 1.5) using a previously described analytical approach [41]. For the other donors (14 males and 12 females; age range: 34–84 years), we did not observe a significant increase in the anti-N levels. No other specific criteria such as number of patients (sample size), sex, clinical or demographic data were used for inclusion.

### 2.3. Generation of Plasmids

The codon-optimized SARS-CoV-2 Spike D614G was amplified without the stop codon with PCR using a previously described pCAGGS-SARS-CoV-2 S D614G construct [45] and fused to GFP with overlapping PCR using amplified linker and C-terminal GFPSpark tag from a pLV-SARS-CoV-2 S C-GFPSpark tag ([46], Sino Biological) and cloned using EcoRI and NotI into this same vector to replace the ancestral S-GFP coding sequences to produce a pLV-SARS-CoV-2 S D614G C-GFPSpark tag. A synonymous mutation was also introduced in the first codon of the Glycine linker to generate an AgeI site to facilitate the cloning of the other S variants. Previously described SARS-CoV-2 S variants (BA.1, BA.2, BA2.12.1 and BA4/5) codon-optimized genes [15,18] were amplified without the stop codon and cloned to replace S into the pLV-SARS-CoV-2 S D614G C-GFPSpark tag described above using EcoRI and AgeI. All constructs were verified by whole-plasmid sequencing (Plasmidsaurus).

### 2.4. Cell Lines 

The cell lines were generated as previously described [46,47]. Briefly, for the generation of CEM.NKr CCR5+ cells stably expressing the SARS-CoV-2 Spike D614G; BA.1; BA.2; BA.2.12.1; or BA.4/5 glycoproteins, transgenic lentiviruses were produced in 293T cells using a third-generation lentiviral vector system. 293T cells were co-transfected using a standard calcium phosphate protocol with two packaging plasmids (pLP1 and pLP2 [48]), an envelope plasmid (pSVCMV-IN-VSV-G [49]) and a lentiviral transfer plasmid coding for a GFP-tagged SARS-CoV-2 D614G; BA.1; BA.2; BA.2.12.1; or BA.4/5 Spike (pLV-SARS-CoV-2 Spike D614G; BA.1; BA.2; BA.2.12.1; or BA.4/5 C-GFPSpark tag) at a ratio of 2:1:1:4, respectively. Two days post-transfection, supernatants containing lentiviral particles were collected, ultracentrifugated and were used to transduce CEM.NKr CCR5+ cells in presence of 5 µg/mL of polybrene. The CEM.NKr CCR5+ cells stably expressing the different SARS-CoV-2 Spikes were sorted using flow cytometry based on Spike expression (GFP+) and CV3-25 staining (1 µg/mL). The CV3-25 antibody is a conformationally independent anti-S2 antibody which can bind and is effective against all known Spike variants [7,50]. This antibody was used to select cells from the different cell lines with a similar expression level of Spike glycoproteins at their surface. The parental CEM.NKr CCR5+ cells (NIH AIDS Reagent Program) and the subsequently generated cell lines were maintained at 37 °C and 5% CO_2_ in RPMI (Thermo Fisher Scientific, Waltham, MA, USA) containing 10% fetal bovine serum (VWR, Radnor, PA, USA) and 100 µg/mL penicillin–streptomycin (Wisent, St. Bruno, QC, Canada).

### 2.5. Anti-Nucleocapsid (N) Assay

Anti-N antibodies were detected using a previously described ELISA [41]. Briefly, recombinant N (Centre National en Électrochimie en Technologies Environnementales Inc., Shawinigan, Quebec, Canada) was used as capture antigen (0.25 µg/mL) in 96-well microplates. Plasma samples were diluted 1:100 and incubated for one hour at room temperature, followed by washing and the addition of anti-human polyvalent IgA+IgG+IgM (H+L)-HRP conjugate as secondary antibody. The plates were incubated once again for one hour at RT followed by washing and addition of 100 µL of 3,3′,5,5′-Tetramethylbenzidine (TMB, ESBE Scientific, Markham, ON, Canada). The colorimetric reaction was stopped after 20 min by the addition of 100 µL of H_2_SO_4_ 1N (Fisher Scientific (Thermo Fisher Scientific), Waltham, MA, USA). The plates were then read within 30 min at 450 nm using a Synergy H1 microplate reader (BioTek, Winooski, VT, USA).

### 2.6. Cell Surface Staining and Flow Cytometry Analysis

For the staining on the CEM.NKr.Spike D614G; BA.1; BA.2; BA.2.12.1; and BA.4/5 cell lines, approximately 300,000 cells were stained in 100 µL of a diluted (1/500) plasma solution at the different time points (W4-VA3, M4-VA3 and W4-VA4). As controls, 5 pre-pandemic plasmas at a dilution of 1/500 and an antibody solution of CV3-25 at 1 µg/mL were used to stain the different cells. These cells were incubated at room temperature (20–25 °C) for 45 min before being washed twice with PBS. Following this, 100 µL of a solution of goat anti-human IgG Alexa-Fluor 647 (Thermo Fisher Scientific, Waltham, MA, USA) secondary antibody (mixed with the viability marker AquaVivid (Thermo Fisher Scientific, Waltham, MA, USA) at a dilution of 1:1000 was added to the cells at a concentration of 2 µg/mL for 20 min at room temperature (20–25 °C). The cells were washed twice in PBS and fixed in a solution of PFA 2% before being passed on a cytometer. All samples were acquired on an LSRII cytometer (BD Biosciences, Mississauga, ON, Canada). Analysis of the staining was performed using FlowJo v10.5.3 (Tree Star, Ashland, OR, USA) by gating on the alive GFP+ population.

### 2.7. Antibody-Dependent Cellular Cytotoxicity (ADCC) Assay

This assay was previously described [46,47]. Briefly, for evaluation of anti-SARS-CoV-2 ADCC activity, parental CEM.NKr CCR5+ cells were mixed at a 1:1 ratio with the different generated cell lines (CEM.NKr.Spike D614G, CEM.NKr.Spike BA.1, CEM.NKr.Spike BA.2, CEM.NKr.Spike BA.2.12.1 or CEM.NKr.Spike BA.4/5). These cells were stained for viability (AquaVivid) and a cellular dye (cell proliferation dye eFluor670; Thermo Fisher Scientific, Waltham, MA, USA) and subsequently used as target cells. PBMCs were stained with another cellular marker (cell proliferation dye eFluor450; Thermo Fisher Scientific, Waltham, MA, USA) and used as effector cells. Stained effector and target cells were mixed at a 10:1 ratio in 96-well V-bottom plates. Plasmas from pre-pandemic, non-recently infected and recently infected individuals at a dilution of 1/500 or monoclonal antibody CV3-25 at 1 µg/mL were added to the appropriate wells. The plates were subsequently centrifuged for 1 min at 300× *g*, and incubated at 37 °C, 5% CO_2_ for 5 h before the addition of 100 µL of a solution of PFA 4% (final concentration: 2% PFA). Since CEM.NKr.Spike D614G; BA.1; BA.2; BA.2.12.1; and BA.4/5 cells express GFP, ADCC activity was calculated using the formula: [(% of GFP + cells in Targets plus Effectors) − (% of GFP + cells in Targets plus Effectors plus plasma or antibody)]/(% of GFP + cells in Targets) × 100 by gating on transduced live target cells. All samples were acquired on an LSRII cytometer (BD Biosciences, Mississauga, ON, Canada) and data analysis was performed using FlowJo v10.5.3 (Tree Star, Ashland, OR, USA).

### 2.8. Virus Neutralization Assay

For this, 293T cells were transfected with the lentiviral vector pNL4.3 R-E− Luc and a plasmid encoding the D614G, BA.1, BA.2, BA.2.12.1 or the BA.4/5 Spike glycoprotein at a ratio of 10:1 to produce SARS-CoV-2 pseudoviruses. Two days post-transfection, cell supernatants were harvested and stored at −80 °C until use. For the neutralization assay, 293T-ACE2 target cells were seeded at a density of 1 × 10^4^ cells/well in 96-well luminometer-compatible tissue culture plates (PerkinElmer, Waltham, MA, USA) 24 h before infection. Pseudoviral particles were incubated with several plasma dilutions (1/50; 1/250; 1/1250; 1/6250; 1/31250) for 1 h at 37 °C and were then added to the target cells followed by incubation for 48 h at 37 °C. Cells were lysed by the addition of 30 µL of passive lysis buffer (Promega, Madison, WI, USA) followed by one freeze–thaw cycle. An LB942 TriStar luminometer (Berthold Technologies, Bad Wildbad, Germany) was used to measure the luciferase activity of each well after the addition of 100 µL of luciferin buffer (15 mM MgSO_4_, 15 mM KH_2_PO_4_ [pH 7.8], 1 mM ATP, and 1 mM dithiothreitol) and 50 µL of 1 mM d-luciferin potassium salt (Prolume, Randolph, VT, USA). The neutralization half-maximal inhibitory dilution (ID_50_) represents the plasma dilution to inhibit 50% of the infection of 293T-ACE2 cells by pseudoviruses.

### 2.9. Statistical Analysis

Symbols represent biologically independent samples from individuals. Statistics were analyzed using GraphPad Prism version 8.0.2 (GraphPad, San Diego, CA, USA). Each dataset was tested for statistical normality and this information was used to apply the appropriate (parametric or nonparametric) statistical test. *p* values < 0.05 were considered significant; significance values are indicated as * *p* < 0.05, ** *p* < 0.01, *** *p* < 0.001, **** *p* < 0.0001; n.s., non-significant.

## 3. Results

### 3.1. Characteristics of the Cohort

To investigate the effect of a recent SARS-CoV-2 infection on humoral responses following a fourth dose of vaccine, we analyzed anti-Spike antibodies induced after the third and fourth doses of mRNA vaccine. Our cohort was composed of 26 non-recently infected individuals (a median of 64.5 years old) and 20 recently infected individuals (a median of 53.5 years old) of which 12/26 and 12/20 were women, respectively. The status of infection (recently vs. non-recently infected) was determined by the increase in anti-N levels between W4-VA3 and M4-VA3 or between M4-VA3 and W4-VA4 (ratio M4-VA3/W4-VA3 and/or W4-VA4/M4-VA3 higher than 1.5) using a previously described analytical approach [41]. The results of the anti-N ELISA are shown in Figure 1B. In our cohort, the Pfizer mRNA monovalent vaccine was used for the first three doses. Among individuals who were not recently infected, the fourth dose was the Pfizer monovalent, Moderna monovalent, Pfizer bivalent (BA.4/5) and Moderna bivalent (BA.1) for 11, 14, 0 and 1 individuals, respectively. Among individuals who were recently infected, the fourth dose was the Pfizer monovalent, Moderna monovalent, Pfizer bivalent (BA.4/5) and Moderna bivalent (BA.1) for 5, 2, 5 and 8 individuals, respectively. For non-recently infected individuals, the median number of days between the third and fourth dose was 135.5 and for infected individuals, this number was 284.5. A median of 23.5 days and 28 days separated the third dose and the first plasma sample (W4-VA3) for non-recently infected individuals and recently infected individuals, respectively. A median of 120 days and 123 days separated the third dose from the second plasma sample (M4-VA3) for non-recently infected individuals and recently infected individuals, respectively. Following the fourth dose, non-recently infected individuals and recently infected individuals waited a median of 28 and 23 days before providing a plasma sample (W4-VA4), respectively. Basic demographic characteristics and detailed vaccination/sampling time points are summarized in Table 1 and Figure 1A. 

### 3.2. CV3-25 Mediates Similar ADCC against All Different Generated Cell Lines

**Figure 2:**
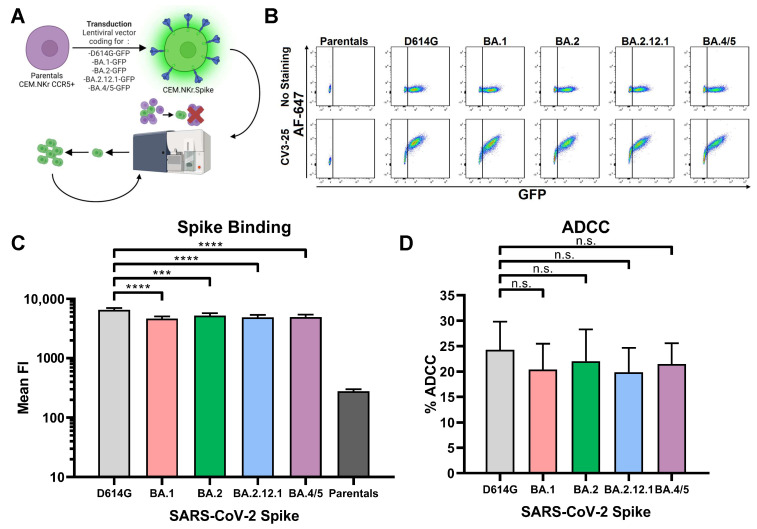
**Characterization of the new cell lines expressing different SARS-CoV-2 Spikes.** (**A**) Schematic representation depicting how the different cell lines were generated. The CEM.NKr CCR5+ parental cells were first transduced with a lentiviral vector coding for one of 5 different Spikes (D614G; BA.1; BA.2; BA.2.12.1; and BA.4/5) fused in their C-terminal with the GFP. The cells were then sorted and amplified multiple times. (**B**) Example of staining (or absence thereof) on the different cell lines by the CV3-25 monoclonal antibody at a concentration of 1 µg/mL. (**C**) Staining with the CV3-25 monoclonal antibody (n = 7) by gating on the GFP+ population (or the GFP- population for the parental cells). Mean FI: Mean Fluorescence Intensity. (**D**) ADCC on the different cell lines (n = 7) by the CV3-25 monoclonal antibody. Statistical significance was evaluated using the appropriate parametric or non-parametric statistical test (unpaired *t*-test or Mann–Whitney test) based on the normal distribution of the data (n.s.: non-significant; ***: *p* < 0.001; ****: *p* < 0.0001). The error bars represent the standard deviation (SD).

To measure ADCC responses against different SARS-CoV-2 variants, we first generated cell lines expressing the D614G, BA.1, BA.2, BA.2.12.1 and BA.4/5 Spikes. The mutations present in the BA.1, BA.2, BA.2.12.1 and BA.4/5 Spikes are shown in Supplemental Table S1. Briefly, we transduced parental CEM.NKr CCR5+ cells with a lentiviral vector coding for the D614G, BA.1, BA.2, BA.2.12.1 and BA.4/5 Spikes fused by their C-terminal domain to GFP. Following this transduction, the cells were amplified and stained with the anti-S2 conformationally independent CV3-25 monoclonal antibody, that efficiently recognizes a highly conserved epitope among all tested SARS-CoV-2 variants so far [7,16,50], before successive rounds of cell sorting (Figure 2A). The resulting cell lines expressed GFP and the Spike at the cell surface, as shown by the CV3-25 staining (Figure 2B). These cell lines expressed similar levels of Spike at their surface, with the exception of the CEM.NKr.Spike D614G cells that expressed slightly higher levels of Spike (Figure 2C). However, no significant differences in CV3-25-mediated ADCC were observed (Figure 2D), thus indicating that the cell lines stably expressing the different Omicron subvariant Spikes could be used to evaluate ADCC mediated by plasma from vaccinated individuals. 

### 3.3. A History of Recent SARS-CoV-2 Infection Significantly Enhances Antibody Binding against Omicron Subvariants

**Figure 3:**
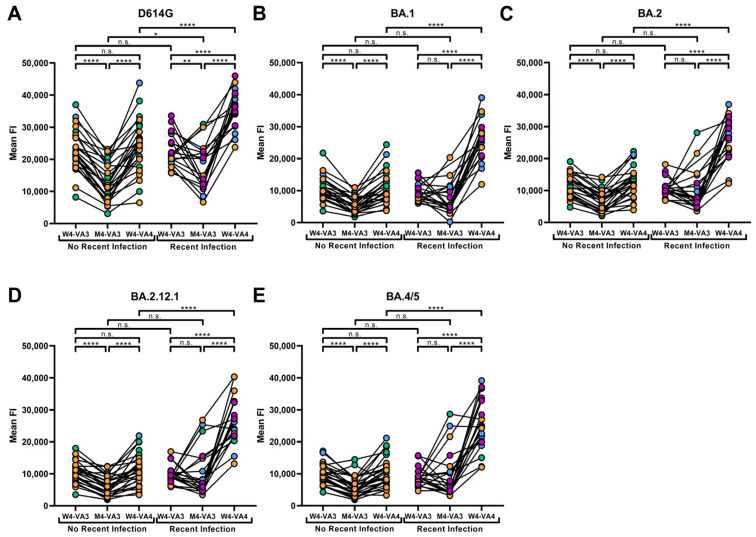
**Spike recognition by plasma from vaccinated individuals presenting a history of recent infection or not.** Staining with the plasmas of non-recently and recently infected individuals was measured four weeks following the third dose (W4-VA3), four months following the third dose (M4-VA3) and four weeks following the fourth dose (W4-VA4) on the cell lines expressing the (**A**) D614G, (**B**) BA.1, (**C**) BA.2, (**D**) BA.2.12.1 and (**E**) BA.4/5 Spikes by gating on the GFP+ population. Circles filled with orange, green, purple or blue represent data points of individuals who received a fourth dose of Pfizer monovalent, Moderna monovalent, Pfizer bivalent or Moderna bivalent vaccine, respectively. Statistical significance between groups (unpaired *t*-test or Mann–Whitney test) or within groups (paired *t*-test or Wilcoxon test) was evaluated using the appropriate parametric or non-parametric statistical test based on the normal distribution of the data (n.s.: non-significant; *: *p* < 0.05; **: *p* < 0.01; ****: *p* < 0.0001). Mean FI: Mean Fluorescence Intensity.

To evaluate how the third and fourth dose modulate ADCC activity, we collected plasma samples from a cohort of non-recently and recently infected individuals (as determined by an increase in their anti-N levels (Figure 1B)) four weeks following the third dose, four months following the third dose and four weeks following the fourth dose. We first evaluated the capacity of plasma from our cohort (Table 1) to bind the different Spikes. In both groups, we measured high levels of antibodies binding the D614G Spike after the third dose of vaccine (W4-VA3) (Figure 3A, Supplemental Figure S1A–C). For the group without a recent infection, the levels of antibodies strongly decreased 4 months following the third dose of vaccine (M4-VA3), and were significantly increased by the fourth dose (W4-VA4) to the same levels as after the third dose (Figure 3A, Supplemental Figure S1A–C). Individuals with a history of recent infection presented an overall decrease in anti-Spike antibodies at M4-VA3. However, this level was slightly higher than in the non-recently infected group. This difference in antibody levels between the two groups became more pronounced following the fourth vaccine dose (W4-VA4) due to the recent infection of all the individuals of this group (Figure 3A, Supplemental Figure S1A–C). We note, however, that the recently infected individuals received in a larger proportion the bivalent mRNA vaccine compared to the non-recently infected individuals. 

We next evaluated plasma binding using cell lines expressing the Omicron Spike (BA.1) and three subvariants (BA.2, BA.2.12.1 and BA.4/5). As expected, a significant decrease in antibody binding was observed against these Omicron subvariants compared to the D614G Spike at every timepoint (Supplemental Figure S1A–C). However, the general pattern of Spike recognition was similar to that observed with the D614G Spike (Figure 3A–E). The anti-Spike antibodies in individuals that were not infected recently significantly decreased between 4 weeks and 4 months following the third dose but were restored after the fourth dose (Figure 3B–E). In comparison, in individuals which were recently infected, levels of anti-Spike antibodies did not significantly decrease between 4 weeks and 4 months following the third dose (Figure 3B–E). Moreover, we observed a significant increase in the Omicron Spikes recognition following the fourth dose, higher than what was measured in the group of individuals without a history of recent infection (Figure 3B–E, Supplemental Figure S1C). Interestingly, only recently infected individuals had higher antibody levels after the fourth dose compared to the third (Figure 3A–E), suggesting that a vaccine boost elicits higher humoral responses in individuals with a history of recent infection.

### 3.4. A Recent SARS-CoV-2 Infection Significantly Enhances ADCC and Neutralization against Omicron Subvariants

**Figure 4:**
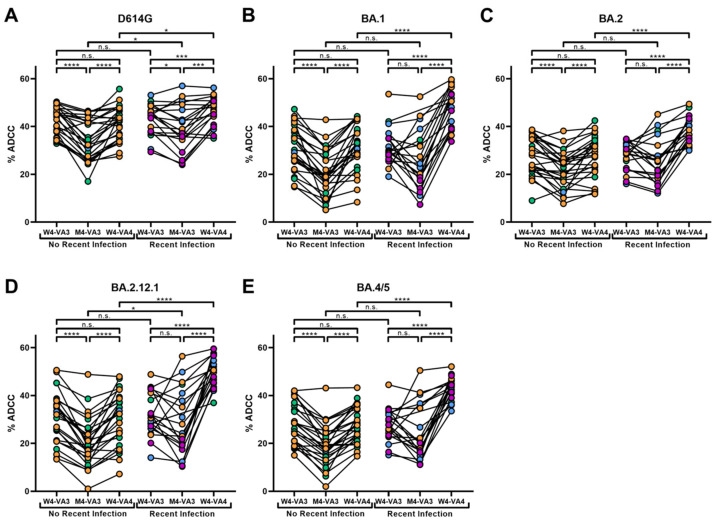
**ADCC mediated by plasma from vaccinated individuals presenting a history of recent infection or not.** ADCC with the plasmas of non-recently and recently infected individuals was measured four weeks following the third dose (W4-VA3), four months following the third dose (M4-VA3) and four weeks following the fourth dose (W4-VA4) on the cell lines expressing the (**A**) D614G, (**B**) BA.1, (**C**) BA.2, (**D**) BA.2.12.1 and (**E**) BA.4/5 Spikes. Circles filled with orange, green, purple or blue represent data points of individuals who received a fourth dose of Pfizer monovalent, Moderna monovalent, Pfizer bivalent or Moderna bivalent vaccine, respectively. Statistical significance between groups (unpaired *t*-test or Mann–Whitney test) or within groups (paired *t*-test or Wilcoxon test) was evaluated using the appropriate parametric or non-parametric statistical test based on the normal distribution of the data (n.s.: non-significant; *: *p* < 0.05; ***: *p* < 0.001; ****: *p* < 0.0001).

Antibodies are complex pleiotropic molecules which can mediate multiple activities including neutralizing viral particles and mediating Fc-effector functions. We measured the ADCC capacity of plasma samples from our cohort of non-recently and recently infected individuals using the cell lines stably expressing the D614G, BA.1, BA.2, BA.2.12.1 or BA.4/5 Spikes. Briefly, in our assay, we mixed our parental CEM.NKr cells and one of our newly engineered CEM.NKr.Spike cell lines. Following this, we added PBMCs as effector cells and the different plasma samples from our cohort. Plasma samples were incubated with the cells for 5 h, as previously described [38,46,47]. Four weeks following the third vaccine dose, both groups presented similar ADCC responses against D614G Spike expressing cells (Figure 4A). Four months after the third dose, decreases in ADCC were more pronounced in the non-recently infected group than the recently infected one (Figure 4A, Supplemental Figure S1E) and the administration of a fourth dose significantly enhanced ADCC responses in both groups (Figure 4A). However, slightly higher ADCC levels were measured in the recently infected group, compared to the other group (Figure 4A, Supplemental Figure S1F). 

We then measured ADCC responses against cells expressing the Omicron Spike (BA.1) or three Omicron subvariants (BA.2, BA.2.12.1 and BA.4/5). As observed for the D614G Spike, 4 weeks after the third dose of vaccine, both groups had similar ADCC responses against cells expressing the Omicron subvariants Spike (Figure 4B–E, Supplemental Figure S1D). In non-recently infected individuals, ADCC levels significantly decreased at M4-VA3, and the fourth dose boosted the ADCC to a similar level than after the third dose (Figure 4B–E). In contrast, in recently infected individuals, we did not observe a significant decrease in ADCC four months following the third dose, with the fourth dose greatly enhancing ADCC (Figure 4B–E, Supplemental Figure S1F). Interestingly, ADCC was higher in recently infected individuals compared to non-recently infected individuals after the fourth dose (Figure 4A–E, Supplemental Figure S1F). We also noted a significantly lower ADCC against the Omicron subvariants at both time points following the third dose (W4-VA3 and M4-VA3) compared to D614G (Figure 4A–E, Supplemental Figure S1D–E). Overall, these data show that ADCC levels following the fourth dose are significantly enhanced by a recent SARS-CoV-2 infection.

**Figure 5:**
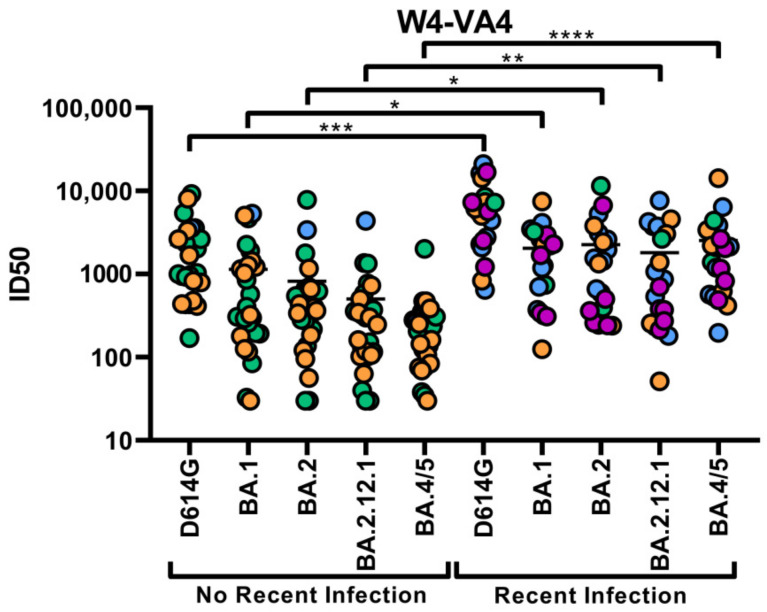
**Neutralization capacity of the plasmas following a fourth vaccine dose.** Neutralization capacity of plasmas from non-recently and recently infected individuals was tested against pseudoviruses bearing either the D614G, BA.1, BA.2, BA.2.12.1 or BA.4/5 Spike glycoproteins 4 weeks following the fourth dose. Neutralization half-maximal inhibitory plasma dilution (ID_50_) values were determined using a normalized non-linear regression using GraphPad Prism software. Circles filled with orange, green, purple or blue represent data points of individuals who received a fourth dose of Pfizer monovalent, Moderna monovalent, Pfizer bivalent or Moderna bivalent vaccine, respectively. Statistical significance between groups (unpaired *t*-test or Mann–Whitney test) was evaluated using the appropriate parametric or non-parametric statistical test based on the normal distribution of the data (n.s.: non-significant; *: *p* < 0.05; **: *p* < 0.01; ***: *p* < 0.001; ****: *p* < 0.0001).

Since the neutralizing capacity of plasma from vaccinated/infected individuals was reported to be compromised against Omicron subvariants [13,14,15,16,18,19,20,21,22,23,24,25], we measured neutralization using pseudoviral particles bearing the different Spikes (Figure 5). Briefly, pseudoviruses bearing the different Spikes were produced in 293T cells. These pseudoviruses were incubated with several plasma dilutions for one hour at 37 °C. Following this, the pseudoviruses were incubated for 48 h on 293T-ACE2 target cells. The cells were then lysed and the neutralization half-maximal inhibitory dilutions (ID_50_) were calculated. While we observed that all Omicron subvariants were more resistant to neutralization than the D614G Spike in both groups, we also observed that recently infected individuals had significantly higher levels of neutralization against all pseudoviral particles (Figure 5). Overall, these results show that neutralization is more affected by changes in the Spike than ADCC (Supplemental Figure S2A,B).

## 4. Discussion

With the continuous advent of new Omicron subvariants presenting an increasing ability to evade neutralization [13,14,15,16,18,19,20,21,22,23,24,25], concerns about vaccine efficacy have increased. To boost antibody and neutralizing responses, the administration of a third and a fourth vaccine dose was recommended by multiple Public Health Authorities worldwide. Nevertheless, compared to the ancestral strain, the new Omicron subvariants remain more resistant to neutralization even after four doses [16,18,24]. While neutralization is an important part of the immune response, other antibody functions such as Fc-effector functions likely help to combat the infection. To study the role of ADCC during SARS-CoV-2 infections, we developed an assay to measure ADCC responses against cells expressing the Spike of the virus [46,47]. We found that three weeks after a single dose of BNT162b2 vaccine, antibodies with high ADCC and low neutralization activity were induced in naïve individuals [34]. Interestingly, a single dose of BNT162b2 vaccine induced >90% effectiveness beginning two weeks (at a time when minimal neutralization was detected) after the first dose of the vaccine [35,51]. These findings further suggested a role of non-neutralizing functions in preventing and clearing the SARS-CoV-2 infection. To address this question, we analyzed plasma samples from non-recently and recently infected individuals who received a third and fourth vaccine dose. The individuals of our cohort were separated according to their history of recent infection as described in the method section and Figure 1. Both groups had significant binding and ADCC capacity against the D614G strain following the third and fourth doses (with slightly more binding and ADCC in the recently infected individuals after the fourth dose). When measuring the ADCC responses against BA.1, BA.2, BA.2.12.1 and BA.4/5, we noticed significant decreases in the ADCC responses before the fourth dose for both groups compared to D614G. However, following the fourth dose, recently infected individuals had markedly higher ADCC than non-recently infected individuals, who had only minimal increases in their ADCC responses. In addition, differences in ADCC levels between Omicron subvariants and D614G and between recently and non-recently infected individuals seem less pronounced than for neutralization, suggesting that Fc-effector functions are more robust and stable than the neutralizing responses against different SARS-CoV-2 variants. Considering that in K18-hACE2 mice, Fc-effector functions alone delayed virus spread, neuroinvasion, and death [40] and that Fc-effector functions of plasma with low compromised neutralization provided a second line of defense against SARS-CoV-2 infection [52], the relative stability of Fc-effector functions might explain why vaccines still provide protection against severe disease and death even when neutralization is compromised. Our results suggest that Fc-effector functions plays a role in protection against severe disease/death and clearance of Omicron subvariant infections even with compromised neutralization.

## 5. Conclusions

Overall, our results show that a recent infection in addition to a fourth vaccine dose (i.e., hybrid immunity) provides much better antibody binding and Fc-effector functions than vaccination alone. Furthermore, while differences in ADCC were observed between non-recently infected and recently infected individuals, the ADCC capacity of these two groups was more stable than their neutralizing response against the tested Omicron subvariants, thus supporting the important role of Fc-effector functions in vaccine efficacy.

## Figures and Tables

**Table 1:** **Characteristics of the cohort.** Plasma samples were recovered at an average of 3 to 4 weeks following the third dose (W4-VA3), 4 months following the third dose (M4-VA3) and 3 to 4 weeks following the fourth dose (W4-VA4). ^a^ Values displayed are numbers. ^b^ Values displayed are medians, with ranges in parentheses. ^c^ Values displayed are medians with standard deviation in parentheses (*n.s.*: non-significant; *: *p* < 0.05; **: *p* < 0.01; ***: *p* < 0.001; ****: *p* < 0.0001).

		Entire Cohort	Non-Recently Infected	Recently Infected
Number (*n*) ^a^		46	26	20
Age ^b^ ***		58.5 (24–84)	64.5 (34–84)	53.5 (24–67)
Sex ^a^ *n.s.*	Female (*n*)	24	12	12
	Male (*n*)	22	14	8
Days between the third and fourth doses ^b^ ****		204.5 (101–330)	135.5 (101–271)	284.5 (142–330)
Fourth dose (*n*) ^a^	Pfizer Monovalent *n.s.*	16	11	5
	Moderna Monovalent **	16	14	2
	Pfizer BA.4/5 *	5	0	5
	Moderna BA.1 **	9	1	8
Days between the third dose and sample W4-VA3 ^c^ *n.s.*		25 (6)	23.5 (5)	28 (6)
Days between the third dose and sample M4-VA3 ^c^ *n.s.*		121 (18)	120 (22)	123 (11)
Days between the fourth dose and sample W4-VA4 ^c^ *		28 (13)	28 (15)	23 (7)

## Data Availability

Further information, data reported in this paper, and requests for resources and reagents should be directed to and will be fulfilled by the lead contact, Andrés Finzi (andres.finzi@umontreal.ca), upon request.

## Supplementary Materials

The following supporting information can be downloaded at: https://www.mdpi.com/article/10.3390/v15061274/s1; Table S1: Mutations in the Spikes of BA.1, BA.2, BA.2.12.1 and BA.4/5; Figure S1: Comparison between antibody binding and ADCC between the D614G Spike and the Omicron subvariant Spikes at each timepoint; Figure S2: Fold decrease comparison (against D614G) between neutralization and ADCC after the fourth vaccine dose.
