# Supplementary material for: A Recent SARS-CoV-2 Infection Enhances Antibody-Dependent Cellular Cytotoxicity against Several Omicron Subvariants following a Fourth mRNA Vaccine Dose
Source: Viruses. 2023 May 29;15(6):1274. doi: 10.3390/v15061274 (PMC10302413; doi:10.3390/v15061274)
Supplement: Supplementary file 1 [file viruses-15-01274-s001.zip › viruses-2384754-supplementary.pdf]

Supplemental Table S1. List of mutations of the Omicron variants studied

Variant	Mutations
D614G	D614G
BA.1	A67V, Δ69-70, T95I, G142D/Δ143-145, Δ211/L212I, ins214EPE, G339D, S371L, S373P, S375F, K417N, N440K, G446S, S477N, T478K, E484A, Q493R, G496S, Q498R, N501Y, Y505H, T547K, D614G, H655Y, N679K, P681H, N764K, D796Y, N856K, Q954H, N969K, L981F
BA.2	T19I, L24S, Δ25-27, G142D, V213G, G339D, S371F, S373P, S375F, T376A, D405N, R408S, K417N, N440K, S477N, T478K, E484A, Q493R, Q498R, N501Y, Y505H, D614G, H655Y, N679K, P681H, N764K, D796Y, Q954H, N969K
BA.2.12.1	T19I, LPPA24S, G142D, V213G, G339D, S371F, S373P, S375F, T376A, D405N, R408S, K417N, N440K, L452Q, S477N, T478K, E484A, Q493R, Q498R, N501Y, Y505H, D614G, H655Y, N679K, P681H, S704L, N764K, D796Y, Q954H, N969K
BA.4/5	T19I, LPPA24S, Δ69-70, G142D, V213G, G339D, S371F, S373P, S375F, T376A, D405N, R408S, K417N, N440K, L452R, S477N, T478K, E484A, F486V, Q498R, N501Y, Y505H, D614G, H655Y, N679K, P681H, N764K, D796Y, Q954H, N969K

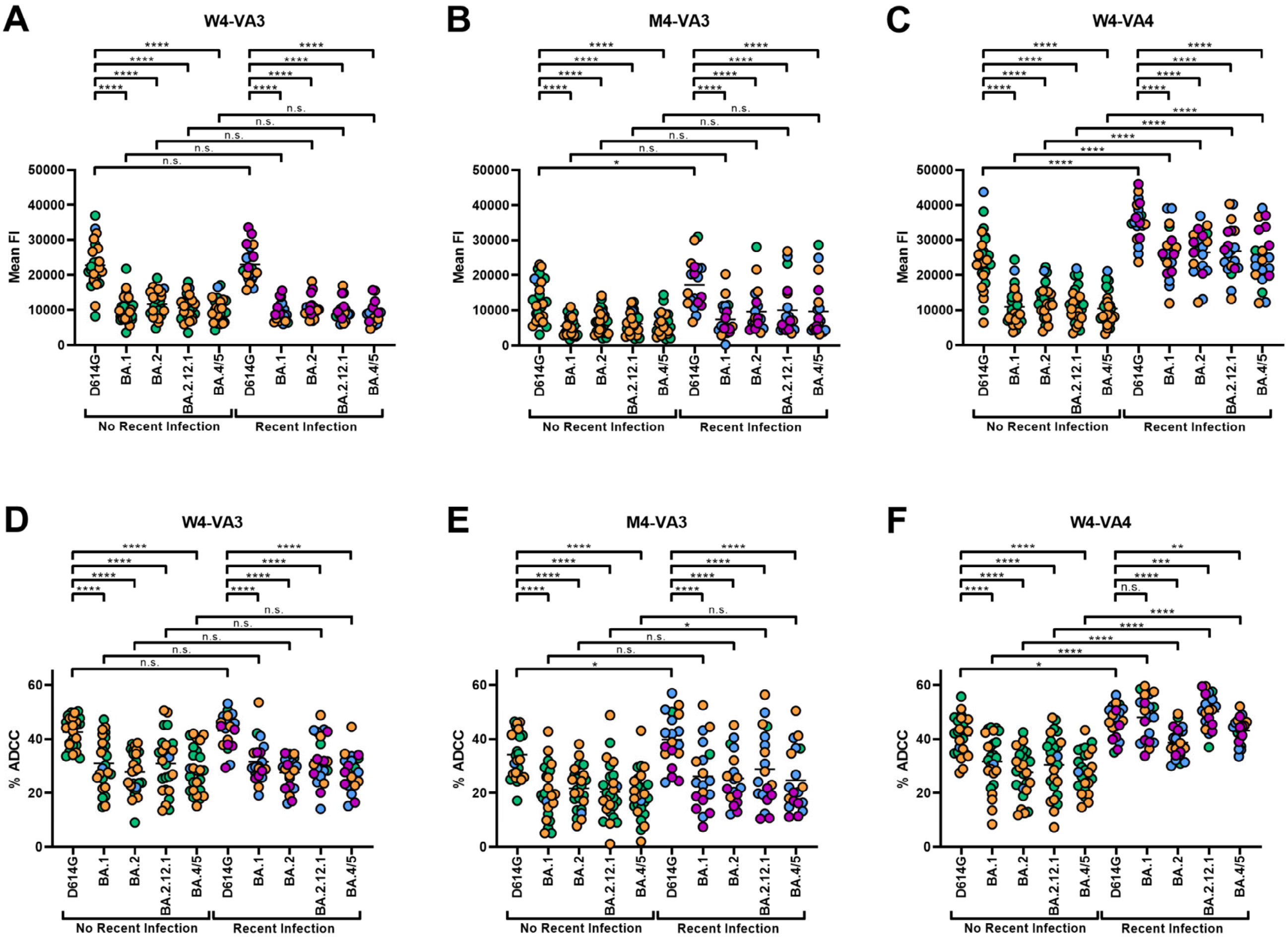

Supplemental Figure S1. Comparison between antibody binding and ADCC between the D614G Spike and the Omicron subvariant Spikes at each timepoint. Antibody binding on the D614G, BA.1, BA.2, BA.2.12.1 and BA.4/5 Spikes of the plasmas of non-recently infected individuals and recently infected individuals at (**A**) 4 weeks following the third dose, (**B**) 4 months following the third dose and (**C**) 4 weeks following the fourth dose. ADCC capacity against D614G, BA.1, BA.2, BA.2.12.1 and BA.4/5 Spike expressing cells of the plasmas of non-recently infected individuals and recently infected individuals at (**D**) 4 weeks following the third dose, (**E**) 4 months following the third dose and (**F**) 4 weeks following the fourth dose. Circles filled with orange, green, purple or blue represent data points of individuals which received a fourth dose of Pfizer monovalent, Moderna monovalent, Pfizer bivalent or Moderna bivalent vaccine, respectively. Statistical significance between groups (unpaired t-test or Mann-Whitney test) or within groups (paired t-test or Wilcoxon test) was evaluated using the appropriate parametric or non-parametric statistical test based on the normal distribution of the data (n.s. : non-significant; * : $p < 0.05$; ** : $p < 0.01$; *** : $p < 0.001$; **** : $p < 0.0001$). Mean FI : Mean Fluorescence Intensity.

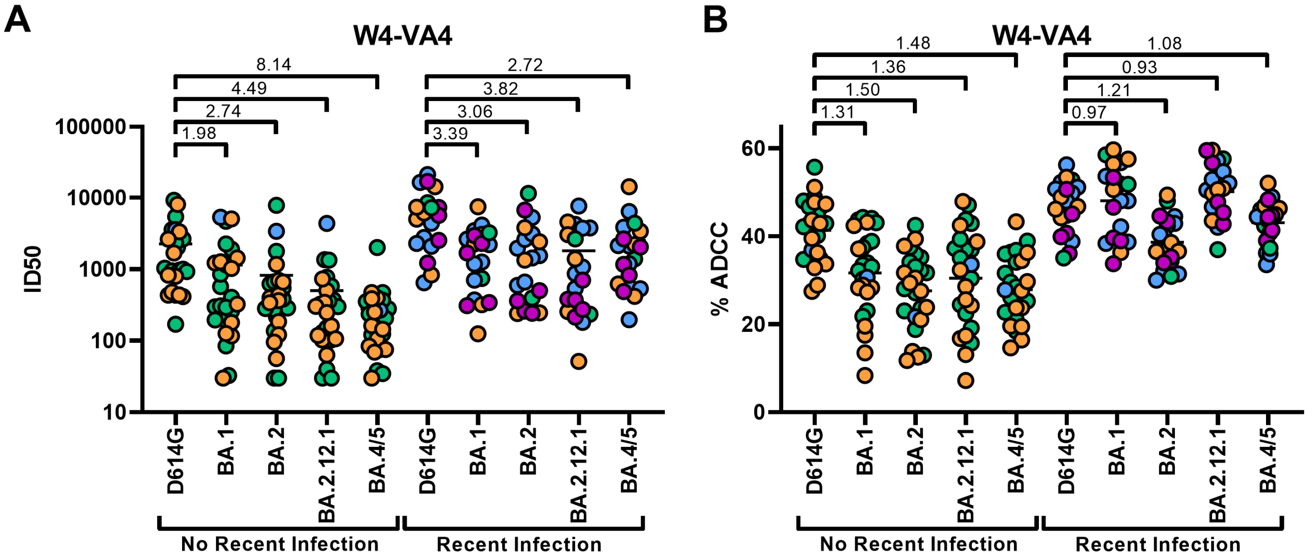

Supplemental Figure S2. Fold decrease comparison (against D614G) between neutralization and ADCC after the fourth vaccine dose. (**A**) Neutralization capacity of plasmas from non-recently and recently infected individuals tested against pseudoviruses bearing either the D614G, BA.1, BA.2, BA.2.12.1 or BA.4/5 Spike glycoproteins 4 weeks following the fourth dose. Neutralization half maximal inhibitory plasma dilution (ID_{50}) values were determined using a normalized non-linear regression using GraphPad Prism software. (**B**) ADCC capacity against D614G, BA.1, BA.2, BA.2.12.1 and BA.4/5 Spike expressing cells of the plasmas of non-recently infected individuals and recently infected individuals at 4 weeks following the fourth dose. Circles filled with orange, green, purple or blue represent data points of individuals which received a fourth dose of Pfizer monovalent, Moderna monovalent, Pfizer bivalent or Moderna bivalent vaccine, respectively. Fold decreases comparison with the D614G Spike are noted in (A) and (B).
